# The Role of Oxidative Stress and Membrane Transport Systems during Endometriosis: A Fresh Look at a Busy Corner

**DOI:** 10.1155/2018/7924021

**Published:** 2018-03-21

**Authors:** Salvatore Giovanni Vitale, Stella Capriglione, Isabel Peterlunger, Valentina Lucia La Rosa, Amerigo Vitagliano, Marco Noventa, Gaetano Valenti, Fabrizio Sapia, Roberto Angioli, Salvatore Lopez, Giuseppe Sarpietro, Diego Rossetti, Gabriella Zito

**Affiliations:** ^1^Unit of Gynecology and Obstetrics, Department of Human Pathology in Adulthood and Childhood “G. Barresi”, University of Messina, Via Consolare Valeria 1, 98125 Messina, Italy; ^2^Department of Obstetrics and Gynecology, Campus Bio-Medico University of Rome, Via Álvaro del Portillo 21, 00128 Rome, Italy; ^3^Department of Medicine, Surgery and Health Sciences, University of Trieste, Ospedale di Cattinara, Strada di Fiume 447, 34149 Trieste, Italy; ^4^Unit of Psychodiagnostics and Clinical Psychology, University of Catania, Via Santa Sofia 78, 95124 Catania, Italy; ^5^Department of Woman and Child Health, University of Padua, Via Giustiniani 3, 35128 Padua, Italy; ^6^Department of General Surgery and Medical Surgical Specialties, University of Catania, Via Santa Sofia 78, 95124 Catania, Italy; ^7^Unit of Gynecology and Obstetrics, Desenzano del Garda Hospital, Section of Gavardo, Via A. Gosa 74, 25085 Gavardo, Italy; ^8^Department of Obstetrics and Gynecology, Institute for Maternal and Child Health-IRCCS “Burlo Garofolo”, Via dell'Istria 65/1, 34137 Trieste, Italy

## Abstract

Endometriosis is a condition characterized by the presence of endometrial tissue outside the uterine cavity, leading to a chronic inflammatory reaction. It is one of the most widespread gynecological diseases with a 10–15% prevalence in the general female population, rising up to 30–45% in patients with infertility. Although it was first described in 1860, its etiology and pathogenesis are still unclear. It is now accepted that inflammation plays a central role in the development and progression of endometriosis. In particular, it is marked by an inflammatory process associated with the overproduction of an array of inflammatory mediators such as prostaglandins, metalloproteinases, cytokines, and chemokines. In addition, the growth and adhesion of endometrial cells in the peritoneal cavity due to reactive oxygen species (ROS) and free radicals lead to disease onset, its ensuing symptoms—among which pain and infertility. The aim of our review is to evaluate the role of oxidative stress and ROS in the pathogenesis of endometriosis and the efficacy of antioxidant therapy in the treatment and mitigation of its symptoms.

## 1. Introduction

Endometriosis is a chronic gynecological disorder defined by the presence of endometrial tissues outside the uterine cavity; these lesions encompass glands and stroma that respond to local, exogenous, and endogenous hormones [1]. It affects about 10–15% of all women within the reproductive age group and has a significant impact on their quality of life and psychological well-being [2–7]. The prevalence of women with pelvic pain ranges from 30 to 45% of the infertile population [1, 8]. Nevertheless, since diagnosis necessitates surgical confirmation, the factual prevalence of the disease is most likely underestimated. There are three different phenotypes of endometriosis, graded from the least to most severe: (1) superficial peritoneal endometriosis (SUP), (2) ovarian endometrioma (OMA), and (3) deeply infiltrating endometriosis (DIE)—the latter being the most aggressive form, characterized by the involvement of the muscularis propria, irrespective of the anatomical location [9–12].

The etiology of endometriosis is complex and indeed still poorly understood. Various theories have been postulated, such as menstrual blood regurgitation, persistent Müllerian duct abnormality, and coelomic metaplasia [13, 14]. The most commonly accepted theory regarding the origin of endometriosis was first postulated by Sampson in 1927. In his work, Sampson described the elements generally present in this condition: retrograde menstruation, viable cells within retrograde menstruation, and the implantation of viable endometrial tissue within the peritoneum [15]. Retrograde menstruation is the backflow of menstrual blood into the peritoneal cavity through the fallopian tubes. Interestingly, retrograde menstruation is not a unique phenomenon to endometriosis and occurs in most women [16]. Normally, the immune system will eliminate these cells, preventing their implantation in the peritoneal cavity. In failing to do so, the patient develops endometriosis. It is now accepted that inflammation plays a central role in the development and progression of endometriosis [17–19]. In particular, the underlying condition is an inflammatory process leading to the overproduction of a wide range of inflammatory mediators, that is, prostaglandins, metalloproteinases, cytokines, and chemokines [20, 21]. Moreover, reactive oxygen species (ROS) and free radicals favor the growth and adhesion of endometrial cells in the peritoneal cavity and consequently disease onset, its related symptoms, pain, and infertility [22–24]. The aim of this review is to evaluate the role of oxidative stress in endometriosis.

## 2. Materials and Methods

The aim of this review is to evaluate the role of oxidative stress in women presenting with endometriosis. Electronic database searches were conducted (MEDLINE, Scopus, Embase, and ScienceDirect) to retrieve relevant studies conducted over the last 20 years. The search terms used were as follows: “endometriosis,” “oxidative stress endometriosis,” “endometriosis,” “infertility,” “infertility in endometriosis,” “oocyte quality,” and “In Vitro Fertilization (IVF).” We only included manuscripts in English. In addition, the references of all articles retrieved were examined to identify studies that had not been identified by electronic searches.

The electronic search and eligibility of the studies were independently evaluated by five coauthors (V.L.L.R., G.V., F.S., G.S., and D.R.). Discrepancies were settled by four different coauthors (I.P., A.V., M.N., and R.A.). Two authors (S.G.V. and G.Z.) independently evaluated both inclusion criteria and study selection. Lastly, additional divergences were evaluated by a third external reviewer (A.S.L.). Data abstraction was completed by two independent investigators (S.C. and S.L.), each of whom independently extracted the data from each single study. The data retrieved from eligible studies were extracted without modifying the original data.

## 3. Results

### 3.1. Oxidative Stress (OS)

Oxidative stress (OS) develops as a consequence of an imbalance between the generation of free radicals and the scavenging capacity of antioxidants. Free radicals are defined as any species with one or more unpaired electrons in the outer orbit [25]. There are two types of free radicals: reactive oxygen species (ROS) and reactive nitrogen species (RNS). The main free radicals are the superoxide radical, hydrogen peroxide, hydroxyl, and singlet oxygen radicals. ROS are intermediate products of normal oxygen metabolism. Oxygen is required to support life; however, its metabolites can alter cell functions and/or endanger cell survival [26, 27]. Almost all major classes of biomolecules—including lipids, proteins, and nucleic acids—are potential targets for ROS. Hydroxyl radicals are the best-known reactive free radical species; they are able to react with a wide range of cell constituents, among which amino acid residues, purine, and pyrimidine DNA bases, and attack membrane lipids to trigger a free radical chain reaction known as lipid peroxidation. Therefore, ROS must be continuously inactivated to preserve the least amount needed to retain normal cell function. Enzymatic and nonenzymatic antioxidant systems scavenge and deactivate excessive free radicals, in an attempt to prevent cell damage. The dietary intake of nonenzymatic antioxidants, that is, manganese, copper, selenium and zinc, beta-carotenes, vitamin C, vitamin E, taurine, hypotaurine, and group B vitamins, is all able to influence the body's complex antioxidant system [28]. On the other hand, the body also produces several antioxidant enzymes such as catalase, superoxide dismutase, glutathione reductase, and glutathione peroxidase, as well as molecules like glutathione and nicotinamide adenine dinucleotide (NADH). The glutathione produced by the cell has a crucial role in maintaining the normal balance between oxidation and antioxidation. NADH is known to play an antioxidant role in biological systems, through its high reactivity with some free radicals, its high intracellular concentrations, and its remarkable power to reduce all biologically active compounds [29]. Any disruption in the balance between ROS production and antioxidant defense generates higher ROS levels, which might lead to OS and the consequent harmful effects. OS is implicated as a major player in the pathophysiology of endometriosis [30].

### 3.2. Endometriosis and Oxidative Stress

Murphy et al. were one of the first groups to highlight the active role played by OS in the pathogenesis and development of endometriosis [30].

OS results from an imbalance between ROS and antioxidants. ROS molecules are characterized by an unpaired electron and stabilize themselves by extracting electrons from different molecules in the body, such as lipids, nucleic acids, and proteins. Antioxidants are a defense mechanism created by the body to neutralize ROS. Serving as signaling molecules, ROS modify reproductive processes such as tubal function, oocyte maturation, and folliculogenesis [27].

Van Langendonckt et al. described a positive correlation between prevention of endometriosis onset in rabbits and increase antioxidant levels. They also found higher levels of ROS release by macrophages, higher peritoneal levels of oxidized low-density lipoproteins and their by-products, an altered expression of endometrial prooxidant and antioxidant enzymes, and the consumption of peritoneal fluid vitamin E [31].

Retrograde menstruation seems to be associated with highly prooxidant factors—that is, heme and iron—within the peritoneal cavity, in addition to apoptotic endometrial cells known to induce OS. The free or catalytic iron induces ROS production through a Fenton reaction, thereby inducing OS [32]. Iron release results from the metabolism of hemoglobin and heme by macrophages. Recent studies have provided evidence of an iron overload in various components of the peritoneal cavity of affected patients, such as peritoneal fluid, macrophages, and endometriotic lesions, strongly suggesting a disruption in iron homeostasis within the peritoneal cavity [33, 34].

Alizadeh et al., in a case-control study conducted in 2015, observed that serum iron level in patients with endometriosis was significantly higher than that in the control group [35]. Severe hemolysis occurring during retrograde menstruation, a defective or overwhelmed peritoneal disposal system in the presence of increased menstrual reflux, induced an iron overload in the peritoneal environment, which in turn caused the attachment and growth of endometrial cells or fragments. These iron stores might have numerous cytotoxic effects; indeed, they could disrupt the balance between free radical production and antioxidant defense, leading to a causal role of OS in the pathogenesis of endometriosis [36]. Iron toxicity might catalyze the production of an array of free radical damaging species, inducing the deregulation of cellular processes, cell dysfunction, and apoptosis or necrosis through lipid peroxidation, protein, and DNA damage [37–39].

There is evidence that the peritoneal fluid (PF) of women with endometriosis is characterized by high lipoprotein levels, particularly low-density lipoproteins (LDL) that generate oxidized lipid components in a macrophage-rich inflammatory milieu [40]. F2-isoprostanes are a complex family of compounds generated by the nonenzymatic peroxidation of arachidonic acid [41] on cell membranes [42] and LDL particles [43]. Various studies have documented increased 8-iso-prostaglandin and a specific biomarker of lipid peroxidation in vivo [44–46]. The measurement of F2-isoprostanes is the most reliable approach to assess OS status in vivo, and the products of the isoprostane pathway have been found to exert strong biological actions, possibly acting as pathophysiological mediators of the disease [44]. The 8-iso-PGF_2*α*_ not only acts as a vasoconstrictor, but has also been shown to promote mitogenesis and cell adhesion of monocytes and polymorphonuclear cells to endothelial cells and to induce endothelial cell necrosis [47]. Polak et al. observed higher PF 8-OHdG and 8-isoprostane concentrations in patients in the advanced stages of endometriosis, laparoscopically and histopathologically confirmed, compared with patients with simple serous and dermoid ovarian cysts [48].

In a large study by Santulli et al., protein OS markers (thiols, advanced oxidation protein products, protein carbonyl, and nitrates/nitrites) were evaluated in the peritoneal fluid on the basis of surgical classification [49]. The markers were significantly higher only in women with deep endometriosis compared with controls (P1/4.001 and P1/4.05, resp.), whereas other forms of endometriosis (peritoneal and ovarian) showed nonstatistically significant increases. These authors failed to find any difference in protein OS markers between women with peritoneal or ovarian endometriosis and control subjects. Moreover, the fact that the control group included women who had undergone surgery for benign gynecological conditions possibly associated with altered peritoneal protein OS markers is also one of the limitations of the study, which may have led to a bias, as acknowledged by the authors themselves [49] (Table 1).

Recent studies have investigated the role of the immune system and oxidative stress in the development of endometriosis [50]. In particular, some women with endometriosis seem to have an inefficient cleansing mechanism, possibly attributable to a failure of the cellular and humoral immune response whose role is to inhibit the implantation of ectopic endometrial tissue [51].

It has been hypothesized that natural killer (NK) cells may subserve this function. NK cells are the effector cells that usually recognize and destroy tumor cells, virus-infected host cells, and transplanted foreign cell lines. Oosterlynck et al. were the first to provide evidence of decreased NK activity and cytotoxicity against autologous endometrial cells in affected women, which correlated with disease stage [52]. These authors went on to show that peritoneal fluid from women with endometriosis, compared to that from fertile controls, presented significantly more marked NK cell suppressive activity [53]. Other authors also found similar findings in the serum [54] and pelvic fluid [55] of endometriosis patients.

Khan et al. were the first to report that peritoneal lesions involving early, active endometriosis and the adjacent peritoneum harbor large amounts of macrophages possibly involved in the onset of endometriosis [56]. Whereas the role of macrophages should be to clear the peritoneal cavity from endometrial cells, in this condition, they seem to enhance their proliferation by secreting growth factors and cytokines. Against this evidence, whether these immunological alterations induce endometriosis or are its consequence has yet to be unraveled [57–59].

### 3.3. Endometriosis Oxidative Stress and Female Infertility

Despite the wealth of literature on the possible association between endometriosis and infertility, a direct causal relationship has yet to be confirmed [60–63]. Endometriosis is generally considered a cause of infertility, through the mechanical hindrance of sperm–egg encounter caused by adhesions, endometrioma, and pelvic anatomy disruption [50]. However, in less severe cases, where there is no pelvic anatomical distortion, the underlying cause of the decreased fertility is poorly understood. Numerous mechanisms have been proposed to account for fertility impairment. Indeed, endometriosis can cause ovulatory dysfunction, poor oocyte quality, luteal phase defects with implantation failure, and abnormal embryogenesis, all of which may lead to poor fertilization [60, 61].

Marcoux et al., in 1997, and Parazzini, in 1999, in randomized, controlled trials evaluated whether laparoscopic surgery (LPS) enhanced fecundity in infertile women with minimal or mild endometriosis. The authors concluded that even if LPS improved natural fecundity, it was much below the rate expected in fertile women and suggested that other factors might interfere with fertility in early-stage endometriosis [62, 63].

In particular, poor oocyte quality can represent a possible mechanism involved.

Simon et al., in a retrospective study, observed a significantly lower implantation rate (IR) in recipients without endometriosis who received egg from women with endometriosis [64].

Recently, spindle morphology has emerged as a marker of oocyte quality and has led to remarkable technological progress in the ability to visualize this structure.

There are several studies describing the extreme sensitivity of the meiotic spindle to various factors, such as OS. A large body of evidence has shown significant DNA damage and increased anomalies in the microtubules and chromosomes of oocytes incubated with PF from endometriosis patients [65, 66]; as this damage could be prevented supplementing the culture medium with the antioxidant l-carnitine, it has been suggested that impaired oocyte quality in endometriosis may be mediated by oxidative stress [66].

In a prospective study carried out in 2009, Barcelos et al. investigated meiotic spindle and chromosome distribution of in vitro mature (IVM) oocytes from patients with and without endometriosis who underwent IVF. They observed a higher proportion of telophase I oocytes in the endometriosis group and suggested a potential delay or impairment of meiosis I during IVM in the presence of endometriosis, as a consequence of OS [67].

However, the evidence in this field does not allow sound conclusions to be drawn, especially in relation to the different stages of endometriosis and previous treatments received, on oocyte quality.

Oocyte competence depends not only on the quality of the follicular microenvironment, but also on the presence of adequate bidirectional cumulus cell-to-oocyte signaling [68–73].

Cumulus cells (CCs) form a group of closely associated cells surrounding the oocyte in the antral follicle, whereas mural granulosa cells form a lining on the follicular wall.

Granulosa cells (GCs) play an essential role in follicular differentiation, providing optimal conditions for oocyte development, ovulation, fertilization, and subsequent implantation [74]. Moreover, the bidirectional communication between the oocyte and these cells occurs all through follicular development [75–80] and is essential for the acquisition of developmental competence in mammalian oocytes [81–83].

Aromatase is present in GCs and plays a fundamental role in follicle maturation and in determining oocyte quality. In vitro studies using luteinized granulosa cell culture from women with and without endometriosis who underwent ovarian stimulation for IVF showed decreased aromatase activity in the GCs of affected women, which might have induced defects in GCs steroidogenesis and abnormal oocyte functioning [84].

Barcelos et al., in 2015, compared the expression of the aromatase gene (CYP19A1 gene) in CCs of infertile women with and without endometriosis who underwent ovarian stimulation for IVF. The authors demonstrated a lower CYP19A1 expression in CCs of infertile patients with endometriosis compared with infertile women without endometriosis. They also observed a lower number of fertilized oocytes in these women. Based on these results, they hypothesized that the reduced CYP19A1 gene expression in cumulus cells might partly account for the impaired oocyte quality associated with endometriosis [85].

Sanchez et al., in a recent review, evaluated all the aspects that might affect oocyte quality in endometriosis patients. They observed that, compared to other causes of infertility, endometriosis is consistently associated with a reduced number of mature oocytes retrieved, whereas a reduction in fertilization rates is more likely associated with minimal/mild rather than moderate/severe endometriosis [86].

Several studies have shown that women with endometriosis have significantly lower concentrations of antioxidant components such as vitamin A, vitamin C, and selenium, in their follicular fluid (FF) [87–91], as well as lower concentrations of superoxide dismutase (SOD) and vitamin E in both serum and FF [88, 89]. FF as a location for reflecting the metabolic process surrounding a mature oocyte prior to ovulation plays a critical role in the reproductive performance of oocytes [92].

Prieto et al. evaluated vitamins C and E, malondialdehyde, and superoxide dismutase concentrations in plasma and FF. They found lower vitamin C (potent natural antioxidant) values in FF from patients with endometriosis compared with unaffected infertile patients. Furthermore, they found a statistically significant negative correlation between plasma vitamin C levels and the number of oocytes retrieved, number of mature oocytes, and number of fertilized oocytes. The authors concluded that the lower concentrations of plasma vitamin C may reflect an excess consumption aimed at neutralizing ROS. They also found a lower superoxide dismutase concentration in the plasma of endometriosis patients compared with controls, which suggests a decreased antioxidant capacity in patients with endometriosis [89].

This increase in OS status in FF has been recently found also in follicles surrounding an endometrioma, as assessed using proteomics by mass spectrometry, and has been proposed to account for spindle disruption [93]. Similarly, in FF of women with both mild and severe endometriosis, Da Broi et al. found increased follicular 8-hydroxy-2′-deoxyguanosine, an indicator of oxidative DNA damage [94, 95].

Recent studies have reported a role of epigenetic modifications in ROS-induced oxidative stress processes leading to development or progression of aging, pancreatitis, fatty liver disease, stroke, diabetes, cancer, and also endometriosis [96–99].

The altered chromatin conformation represents the basis of epigenetic regulation, since the pattern of gene expression undergoes modifications without altering the genomic sequence. Chromatin conformation can be affected by DNA methylation and posttranslational modifications of histones. OS may partly mediate changes in epigenetic marks such as DNA methylation and histone modifications. Endometriotic cells express variable levels of the DNA methyltransferase enzymes (DNMTs), which introduce and maintain DNA methylation on the C5 position of cytosine in CpG dinucleotides [100]. Abnormal DNA methylation in endometriosis affects the expression of several genes, including homeobox A10 (HOXA10), estrogen receptor beta (ESR2), steroidogenic factor 1 (NR5A1), and aromatase (CYP19A1); these, in turn, alter steroid signaling and responsiveness and are critically involved in development and decidualization.

Furthermore, iron oxidation blocks the catalytic activity of the jumonji gene (JMJ, also known as JARID2). The JMJ protein uses Fe^2+^ and *α*KG as cofactors in an oxidative demethylation reaction via hydroxymethyl lysine. Reactive oxygen species oxidize Fe^2+^ to Fe^3+^, thereby attenuating the activity of the JMJ histone demethylases [101]. Hydrogen peroxide also inhibits histone demethylase activity where increased Fe^2+^ rescued this inhibition. Another epigenetic enzyme utilizing Fe^2+^ is members of the ten–eleven translocation (TET) family of hydroxylases. The ten–eleven translocation protein is an active CpG demethylase converting 5-methylcytosine to 5-hydroxymethylcytosine [98]. Members of the TET protein family play a role in the DNA methylation process and in gene activation. Ten–eleven translocation genes (TET1, TET2, and TET3) are downregulated in endometriosis [102, 103]. Ten–eleven translocation–mediated DNA demethylation may act as a protection against oxidative stress [104].

### 3.4. Endometriosis Associated Ovarian Cancer (EAOC)

Many epidemiological studies underline a possible association between endometriosis and invasive epithelial ovarian cancer, on the basis of the high prevalence and incidence of epithelial ovarian cancer in women with endometriosis [105–107].

Endometriosis associated ovarian cancers (EAOCs) represent a subclass of ovarian neoplasms with specific clinical characteristics that include histology, FIGO stage, CA125 levels, patient age, menopausal status at diagnosis, and survival outcomes [108].

Studies suggest that the most EAOCs are endometrioid and clear cell subtypes, with endometriosis found in 30–55% of clear cell cancers and 30–40% of endometrioid ovarian cancers [109–113].

A meta-analysis on 28 studies showed that the standardized incidence ratio (SIR, defined as observed cases/expected cases after adjusted for age) for epithelial ovarian cancer in women with surgical or histologically diagnosed endometriosis was 1.43–8.95, with a 1.34 odds ratio (OR). The prevalence of epithelial ovarian cancer in women with endometriosis was 2.0–17.0%, and the prevalence of endometriosis in women with epithelial ovarian cancer was 3.4–52.6% [114].

In a recent review, Kobayashi described the possible conditions that could result in ovarian cancer consequent to endometriosis [115]. The autoxidation of hemoglobin in the extracellular milieu releases heme and iron, inducing cellular oxidative damage by promoting reactive oxygen species formation; this, in turn, results in DNA damage and mutations (ovarian cancer initiation from endometriosis). On the other hand, persistent antioxidant production could favor a protumoral microenvironment, resulting in cancer progression [115].

Several gene mutations have been identified concurrently in endometriosis lesions and in EAOCs.

Many studies have assessed LOH at 10q23.3 (such as loss of heterozygosity (LOH) and the mutations that lead to the functional inactivation of the phosphatase and tensin homolog (PTEN) tumor suppressor gene located on chromosome 10q23.3) and microsatellite instability (MSI) (leading to the functional inactivation of the PTEN gene) in EAOC.

In 2000, Sato et al. were the first to understand that the inactivation and loss of heterozygosity (LOH) of the tumor suppressor gene PTEN (locus 10q23.3) due to mutations were associated with both endometrioid and clear cell carcinomas in endometrial and ovarian cancers [116].

Subsequent research identified inactivation of PTEN as an early event in the malignant transformation of endometriosis to EAOC, possibly accounting for the development of as many as 14–20% of epithelial ovarian cancers [117, 118].

Two independent studies carried out in 2010 showed that clear cell and endometrioid ovarian cancers were due to somatic mutations in AT-rich interaction domain-1A (ARID1A) [119, 120]. In most cases, ARID1A mutations are either frameshift or nonsense mutations, suggesting their role as tumor suppressor gene, and BAF250a—the protein encoded—is part of a multiprotein SWItch/Sucrose Non-Fermentable (SWI/SNF) chromatin remodeling complex involved in the regulation of cellular processes including differentiation, proliferation, DNA repair, and tumor suppression [121, 122].

ARID1A mutations were found in 46% (55/119) of ovarian clear cell carcinomas, in 30% (10/33) of endometrioid carcinomas, but in none of serous carcinomas. ARID1A mutations and loss of BAF250a expression were both identified in the tumor and in the contiguous atypical endometriosis but could not be detected in distant endometriotic lesions [119].

ARID1A and PIK3CA mutations were particularly important in the clear cell carcinoma subtype of EAOC. Anglesio et al., using whole-genome and targeted deep sequencing, found concurrent ARID1A and PIK3CA mutations in ovarian clear cell carcinoma and in tumor-adjacent and distant endometriotic lesions, independently of cytological atypia [123].

In a study that included 23 clear cell carcinomas with synchronous putative precursor lesions (i.e., endometriosis adjacent to carcinoma, with or without cytological atypia), as many as 43% (10/23) of ovarian clear cell carcinomas and 90% (9/10) of the coexisting endometriotic epithelium adjacent to the clear cell carcinoma presented PIK3CA gene mutations [124].

Loss of ARID1A and PIK3CA was a common finding in 130 cases of ovarian clear cell carcinoma (56.2% and 45.0%, resp.). Loss of ARID1A was quite frequent (76.9%, 20/26) in clear cell carcinoma with concurrent endometriosis. PIK3CA expression was not related to clinical features or survival of clear cell carcinoma. However, loss of ARID1A, along with low-level HNF-1b expression, was common in patients with cancer recurrence and was correlated with late-stage and worse survival outcome [125].

Another study that included 35 pure-type (73.9% with endometriosis) and 11 mixed-type clear cell carcinomas (45.5% with endometriosis) showed that both ARID1A and p53 were mutually altered in pure-type clear cell carcinoma, at immunohistochemical analysis. Altered expression of p53 in these clear cell carcinomas was associated with significantly worse prognosis than in the case of ARID1A (*P* < 0.001) [126].

Catenin (cadherin-associated protein) beta-1 (CTNNB1) mutations are quite peculiar to ovarian endometrioid carcinoma, at variance with other types of ovarian carcinoma. Mutations in exon 3 of the *β*-catenin gene have been identified in 60% (21/35) of endometrioid carcinoma; these mutations have also been detected in the coexisting nonatypical (52.4%) and atypical (73.3%) endometriosis, in most cases with identical single-nucleotide substitutions. Conversely, no evidence of these mutations was found in clear cell carcinomas and the coexisting endometriosis [127].

Using an animal model, Wu et al. demonstrated that inactivation of PTEN and *β*-catenin pathways in the murine surface epithelium resulted in adenocarcinoma formation with similar morphology as human ovarian endometrioid carcinoma [128].

In an interesting report published in 2015, Winarto et al. studied the effects of oxidative stress—purportedly associated with malignant transformation—on the *ARID1A* gene, by assessing its expression in endometriotic, EAOC, and non-EAOC tissue samples, as well as in endometriotic primary cell cultures [129]. They measured oxidative stress through the activity of the antioxidant enzyme manganese dismutase (MnSOD), malondialdehyde (MDA), and *ARID1A* gene expression in tissue samples from patients with endometriosis, EAOC or nonendometriosis-associated ovarian cancer (non-EAOC). In addition, they added H_2_O_2_ to induce OS in cultured cells from patients affected by primary endometriosis and assessed possible alteration of *ARID1A* gene expression based on different H_2_O_2_ concentrations. The results showed that in endometriotic tissue, the expression of *ARID1A* mRNA was lower than that in normal endometrial (control) and non-EAOC tissues, whereas its protein expression was lower in controls but higher than EAOC and non-EAOC tissues. These findings provide evidence of an early decrease in *ARID1A* expression, especially in its mRNA in endometriotic tissue. Furthermore, oxidative stress seems to play a role in the decreased expression of the *ARID1A* protein and mRNA levels in endometriotic cells. Apparently, oxidative stress suppresses *ARID1A* expression in endometriotic cells; conversely, the low *ARID1A* gene activity occurring in endometriosis could increase the susceptibility of these lesions to malignant transformation [129] (Table 2).

### 3.5. Use of Antioxidants in the Treatment of Endometriosis

The identification of OS as a major player in endometriosis pathophysiology has been noted in various studies addressing the influence of OS reduction as treatment goal. To determine the effects of vitamins E and C, as many as 46 women with endometriosis-related pain were given a two-month treatment with vitamin E (1200 IU) and vitamin C (1000 mg) [130]. Vitamin E is a fat-soluble antioxidant that prevents the formation of vitamin E radicals. Vitamin C was also added to this regimen for its action in recycling vitamin E radial to vitamin E. At the end of this randomized controlled trial, 43% of the patients reported a reduction in chronic pelvic pain, suggesting that vitamins E and C administration might lead to a noticeable pain reduction, even in the short term (*P* = 0.0055). Conversely, control patients did not experience any decrease in pain [130]. Santanam et al. attributed the effects of vitamin supplementation to its antioxidative and anti-inflammatory properties, although they did not describe any clear physiological mechanism underlying this effect [130]. Some insight come from Durak et al. who experimentally induced endometriotic cysts in a rat model [131] subsequently treated with differing doses of vitamin C (0.5 mg, 1.25 mg, and 2.5 mg) to determine whether vitamin C supplementation altered lesion volume and weights. At the end of treatment, cystic lesions in the group treated with 2.5 mg vitamin C were significantly reduced in weight and volume [131], suggesting that antioxidants, such as vitamin C, are able to reduce endometriosis symptoms by reducing lesion size.

Mier-Cabrera et al., in a randomized, double-blind trial, treated endometriosis patients with vitamins C and E or a placebo for 6 months and further evaluated the levels of malondialdehyde (MDA) and lipid hydroperoxides (LOOHs) as peripheral OS markers. Significantly decreased levels of MDA and LOOHs were observed after 4 and 6 months, respectively, confirming that vitamins C and E supplementation is associated with a reduction in OS markers in women diagnosed with endometriosis. However, despite OS marker reduction, pregnancy rate did not improve during or after the intervention [132].

Resveratrol (trans-3,5,40-trihydoxystilbene) is a natural polyphenolic flavonoid synthesized by plants following exposure to ultraviolet radiation. Resveratrol is largely present in seeds and the skin of grapes, in mulberries, and in red wine. Amaya et al. evaluated a possible dose-dependent impact on the endometrium. In addition to its antioxidant properties, resveratrol acts as a phytoestrogen, and its estrogen action appears to be related to the concentrations. At low concentrations, it acts agonistically, whereas at high concentrations, it plays an antagonistic role. As endometriosis is an estrogen-dependent disease, high levels of resveratrol were able to reduce xenograft proliferation of human endometrium in mice [133].

Melatonin is another naturally produced hormone with possible powerful effects on endometriotic lesions. Melatonin has several properties; in addition to its action as free radical scavenger, it stimulates antioxidant production and increases the efficacy of the electron chain function [134, 135]. In humans, it is produced in the pineal gland and has been shown to decrease oxidative damage. To understand the role of melatonin, Yilmaz et al. implanted endometriotic lesions in twenty rats and treated ten with melatonin and ten with a saline solution (control). The outcome measures of this study were changes in lesion volume and weight. In the experimental group, lesion volume (*P* < 0.01) and weight significantly decreased (*P* < 0.05), showing that melatonin is able to induce lesion regression [135].

Epigallocatechin-3-gallate (EGCG) is the most abundant polyphenol found in green tea. It has strong antioxidative, antimitotic, and antiangiogenic properties [136]. Leaves of the tea plant *Camellia sinensis*, which contains high nutraceutical values, are employed to prepare green tea. EGCG was demonstrated to affect several carcinogenetic mechanisms such as mutation, cell proliferation, cell invasion, and apoptosis [137]. As some of these mechanisms are common to endometriosis, its effect on this condition has been studied both in vitro and in vivo.

Matsuzaki et al. assessed the in vitro effect of EGCG in endometriosis. Cell samples from 55 endometriosis patients were treated with EGCG and analyzed via RT-PCR, cell proliferation assays, in vitro migration and invasion assays. EGCG significantly reduced proliferation, cell migration, and invasion of endometriotic cells [138]. Although EGCG appears to beneficially affect endometriosis patients, its low bioavailability circumscribed to the ingestion of pure EGCG or green tea consumption limits its therapeutic use [138].

*N*-Acetylcysteine (NAC), the acetylated form of the amino acid cysteine, naturally present in some substances like garlic, exerts a marked antiproliferative action in vitro on cancer cells of epithelial origin [139]. The action of NAC has no role in cell death nor is it due to an unspecific toxic effect, rather it stems from a complex differentiation pathway, including the activation of several molecular mechanisms all converging toward a proliferation-to-differentiation switch that implies decreased cell proliferation and decreased cell locomotory behavior, particularly relevant in endometriosis. In addition, inflammatory protein activity and gene expression are also downregulated by NAC [140, 141]. Overall, NAC emerges as a thiol-containing compound working in the complex framework of redox signaling, and its effects go far beyond a generic antioxidant action [142].

In 2010, in an attempt to extrapolate these in vitro findings to a murine model of endometriosis, Pittalunga et al. provided evidence that NAC treatment helped reduce endometrioma size, at the same time decreasing tissue inflammation and cell invasiveness [143]. A similar effect of NAC in decreasing hydrogen peroxide production and cell proliferation was evidenced in cell and animal models of endometriosis, the result being attributed to the regulation of the extracellular regulated kinase ERK1/2 [144].

In 2013, these same authors carried out an observational cohort study on ovarian endometriosis, in an attempt to compare the evolution of ovarian endometriomas in NAC-treated and untreated control patients, with ultrasound measurements of mean lesion diameter and volume. NAC was administered per os for 3 months at a dosage of 600 mg three times daily on three consecutive days per week [145]. After three months, mean cyst diameter in NAC-treated patients was slightly reduced (−1.5 mm) whereas untreated patients experienced a significant increase (+6.6 mm; *P* = 0.001). Particularly, during NAC treatment, the percentage of cysts that were reduced in size outnumbered those whose size had increased. Twenty-four NAC-treated patients (versus 1 within controls) were able to avoid the scheduled laparoscopy as the cysts had decreased/disappeared and/or the relevant pain decreased (21 cases) or there was a pregnancy (1 case). There were eight pregnancies among NAC-treated patients versus six in untreated patients. The authors therefore concluded that NAC actually represents a simple and effective treatment for endometriosis, devoid of side effects, and suitable for women desiring a pregnancy [145] (Table 3).

## 4. Conclusions

Endometriosis is a chronic disease affecting nearly 10–15% of women of reproductive age, which leads to pain, irregular bleeding, and infertility [146, 147]. The etiology of endometriosis is not clear although general risk factors such as smoking, alcohol use, and low body mass index seem to have a role in its development [1]; in addition, recent studies have identified a possible role for OS and ROS in this condition. In particular, ROS seems to alter endothelial cell permeability and adhesion molecule expression, triggering an inflammatory process. OS substances may contribute to the pathogenesis of endometriosis through the activation of macrophages [47]. Activated macrophages can aggravate oxidative stress conditions through the production of lipid peroxides and other by-products of the reaction between apolipoproteins and peroxides. The sum of these events increases the concentrations of proinflammatory mediators, thus triggering inflammatory conditions in affected women [21–23]. Recent studies have described a cause–effect relationship between epigenetic mechanisms and endometriosis development. In particular, aberrant DNA methylation and histone modification have been associated with an increased risk of endometriosis. Overall, the available literature focuses on the efficacy of antioxidant therapy in the treatment and mitigation of endometriosis.

## Figures and Tables

**Table 1:** Markers of OS in endometriosis patients.

Reference	Type of biospecimen	OS marker
Rong et al. 2002 [40]	Peritoneal Fluid	↑ lipoproteins, particularly low-density lipoprotein (LDL)
Polak et al. 2013 [48]	Peritoneal fluid	↑ 8-iso-PGF_2*α*_ that promotes vasoconstrictor, but has also mitogenesis and cell adhesion
Santulli et al. 2015 [49]	Peritoneal fluid	↑ thiols, advanced oxidation protein products, protein carbonyl, and nitrates/nitrites in deep endometriosis
Jackson et al. 2005 [22]	Peritoneal fluid	↓ antioxidants
		↑ lipid peroxides

**Table 2:** Major genetic alterations/mutations in different stages of endometriosis associated ovarian cancer (EAOC).

Factor	Genetic alteration	Current data
Tumor suppressor genes	PTEN	Phosphatase and tensin homolog is mutated in many cancers, particularly in endometrial and endometrioid ovarian cancer; its inactivation occurs early during tumorigenesis [102]. PTEN somatic mutations are frequently found in endometriotic cysts [101].
	ARID1A	ARID1A mutations are significantly more common in two ovarian cancer subtypes associated with endometriosis (clear cell and endometrioid). Endometriosis synchronous with ovarian cancer presented more frequent mutations in clones derived from endometriosis samples directly adjacent of the tumor than in those from distant endometriotic lesions [104].

DNA repair	hMLH1	hMLH1 corrects errors in DNA replication; hypermethylation of its promoter occurs early in endometriosis malignant transformation [102].

Loss of heterozygosity (LOH)		A trend to increased LOH frequencies has been reported in solitary endometriosis lesions, endometriosis-associated carcinoma, and endometrioid ovarian cancer. Common LOH events can be identified in endometriosis synchronous with ovarian cancer [101].
	ARID1A and PIK3CA	ARID1A and PIK3CA mutations were found in ovarian clear cell carcinoma and in tumor-adjacent and distant endometriotic lesions, regardless of cytological atypia [112–114].
	ARID1A and p53	Both ARID1A and p53 were mutually altered in pure-type clear cell carcinoma at immunohistochemical analysis. Altered expression of p53 in these clear cell carcinomas was associated with significant worse prognosis than that of ARID1A (*P* < 0.001) [115].
	*β*-Catenin	*β*-*Catenin* mutations and overexpression are very common in ovarian endometrioid carcinoma; approximately 50% of endometrioid carcinoma has *β*-catenin alterations [116]. Endometrioid carcinoma containing *β*-*catenin* mutations is low grade and associated with better prognosis. As many as 90% of endometrioid borderline tumors harbor *β*-*catenin* mutations.

**Table 3:** The effect of antioxidative stress agent on endometriosis and the suggested mechanism.

Drugs	Authors	Study type	Effects	Mechanism
Vitamins C and E	Santanam et al. [130]	Human clinical study	Decreased pelvic pain, dysmenorrhea, dyspareunia, PF inflammatory markers, normal T-cell expression and secretion, interleukin-6, and monocyte chemotactic protein-1	↓ oxidatively modified lipoproteins
	Durak et al. [131]	Animal study	Reduced endometriotic cyst volume, weight, growth, and natural killer cell content	Antioxidant and immune stimulator, stimulation of leukocyte functions, enhanced NK function
	Mier-Cabrera et al. [132]	Human clinical study	Decreased OS markers: MDA, lipid hydroperoxides	Neutralizing ROS and RNS

Resveratrol	Amaya et al. [133]	Animal study	Reduced expression of ESR1 and proliferative activity (Ki67), agonist and antagonist of estrogen in low and high concentrations, respectively	↓ ESR1 in endometrial epithelium, action through nongenomic action of alternative estrogen receptors such as GPR30

Epigallocatechin-3-gallate	Matsuzaki and Darcha [138]	In vitro + in vivo	Inhibited cell proliferation, migration and invasion of endometrial tissue, decreased fibrotic markers, prevented progression of fibrosis	↓ transforming growth factor-b1-dependent increase in the mRNA expression of fibrotic markers, inhibited activation of MAPK and Smad signaling pathways in endometrial and endometriotic stromal cells

*N*-acetyl-l-cysteine	Pittaluga et al. [143]	In vitro + animal study	↓ endometrioma mass, reduced immunohistochemical staining of the inflammation-related COX-2 protein, and decreased MMP-9 expression and activity	Switching cell behavior from proliferation toward differentiation and decreased both tissue inflammation and cell invasiveness
	Porpora et al. [145]	Clinical study	↓ endometrioma size, pain reduction, decrease in cell invasive behavior, and ↓ in the inflammatory COX-2	↑ proteins of cell-cell junction complex such as E-cadherin and *β*-catenin
